# Machine learning and statistical inference in microbial population genomics

**DOI:** 10.1186/s13059-025-03775-4

**Published:** 2025-09-27

**Authors:** Samuel K. Sheppard, Nicolas Arning, David W. Eyre, Daniel J. Wilson

**Affiliations:** 1 Ineos Oxford Institute for Antimicrobial Research, Department of Biology, University of Oxford, Oxford, United Kingdom; 2 Big Data Institute, Oxford Population Health, University of Oxford, Oxford, United Kingdom; 3 NIHR Oxford Biomedical Research Centre, Oxford, United Kingdom; 4 NIHR Health Protection Research Unit in Healthcare Associated Infections and Antimicrobial Resistance, University of Oxford, Oxford, United Kingdom; 5 Oxford University Department for Continuing Education, Oxford, United Kingdom

## Abstract

The availability of large genome datasets has changed the microbiology research landscape. Analyzing such data requires computationally demanding analyses, and new approaches have come from different data analysis philosophies. Machine learning and statistical inference have overlapping knowledge discovery aims and approaches. However, machine learning focuses on optimizing prediction, whereas statistical inference focuses on understanding the processes relating variables. In this review, we outline the different aspirations, precepts, and resulting methodologies, with examples from microbial genomics. Emphasizing complementarity, we argue that the combination and synthesis of machine learning and statistics has potential for pathogen research in the big data era.

## Background

Advances in technology and data generation have driven a big data revolution in microbiology, with studies routinely analyzing thousands of whole genome sequences. Datasets generated with ever-increasing volume, variety, and velocity bring tremendous opportunities as well as unique analysis challenges. Inspired by the promise of deeper understanding and driven by high-throughput low-cost DNA sequencing, there are now vast genome libraries of bacterial species approaching one million genomes [1]. Achieving the potential of these resources has required the scaling of conventional statistical methods which face challenges with high-dimensional data, necessitating simplifications and approximations [2]. This is paradoxical, because the vast information content of modern resources should make it easier to glean biological insights about evolutionary origins, transmission dynamics and the genetic basis of phenotypic diversity. Machine learning (ML) approaches offer a potential solution as they can handle very large and heterogeneous datasets [3]. ML is a multidisciplinary pursuit that draws heavily on statistics and computer science. Quantitative approaches to exploiting data underpin both endeavors, but for the purposes of this review we work with the following distinction: statistical inference is a tool for furthering our scientific understanding of the world, while ML is a tool for engineering automatable solutions to prediction, simulation, and pattern recognition.

ML has driven breakthroughs in generative artificial intelligence (AI) including natural language, image, and audio creation [4]. In the biological sciences, ML has surpassed human-engineered solutions to the prediction of 3D protein structures [5], translating nanopore potentials into DNA base calls [6], and discovering antimicrobial peptides from large protein and metagenomic databases [7, 8]. In microbial population genomics, contemporary big data whole-genome approaches often combine statistical inference and ML to answer diverse questions relating to the evolution and epidemiology of infectious diseases [9]. These include predicting future events (e.g., outbreaks), understanding the impact of variables (e.g., virulence and resistance genes), and discovering data patterns (e.g., commonalities in infection risk). Often the best tool for the job is unclear or ambiguous. Here, we provide some perspective on the suitability of statistical inference versus ML for different problems in microbial population genomics by summarizing the approaches and discussing examples.

## Principles of machine learning and statistical inference

ML and statistical inference are tools for modelling often large and complex data that have been numerically encoded into one or more variables, say input features *x* and outcomes *y*. For a comprehensive introduction to the subject, see Murphy [10, 11]. A unifying concept is the *data generating process*, which represents the underlying scientific and sampling processes that led to the data at hand. Both ML and statistics attempt to approximate the data generating process as a mathematical function. Broadly speaking, statistics tends to employ models motivated by a desire to understand underlying *processes*, while ML employs flexible models that can faithfully reproduce observed *patterns*, agnostic to the underlying process.

**Fig. 1:**
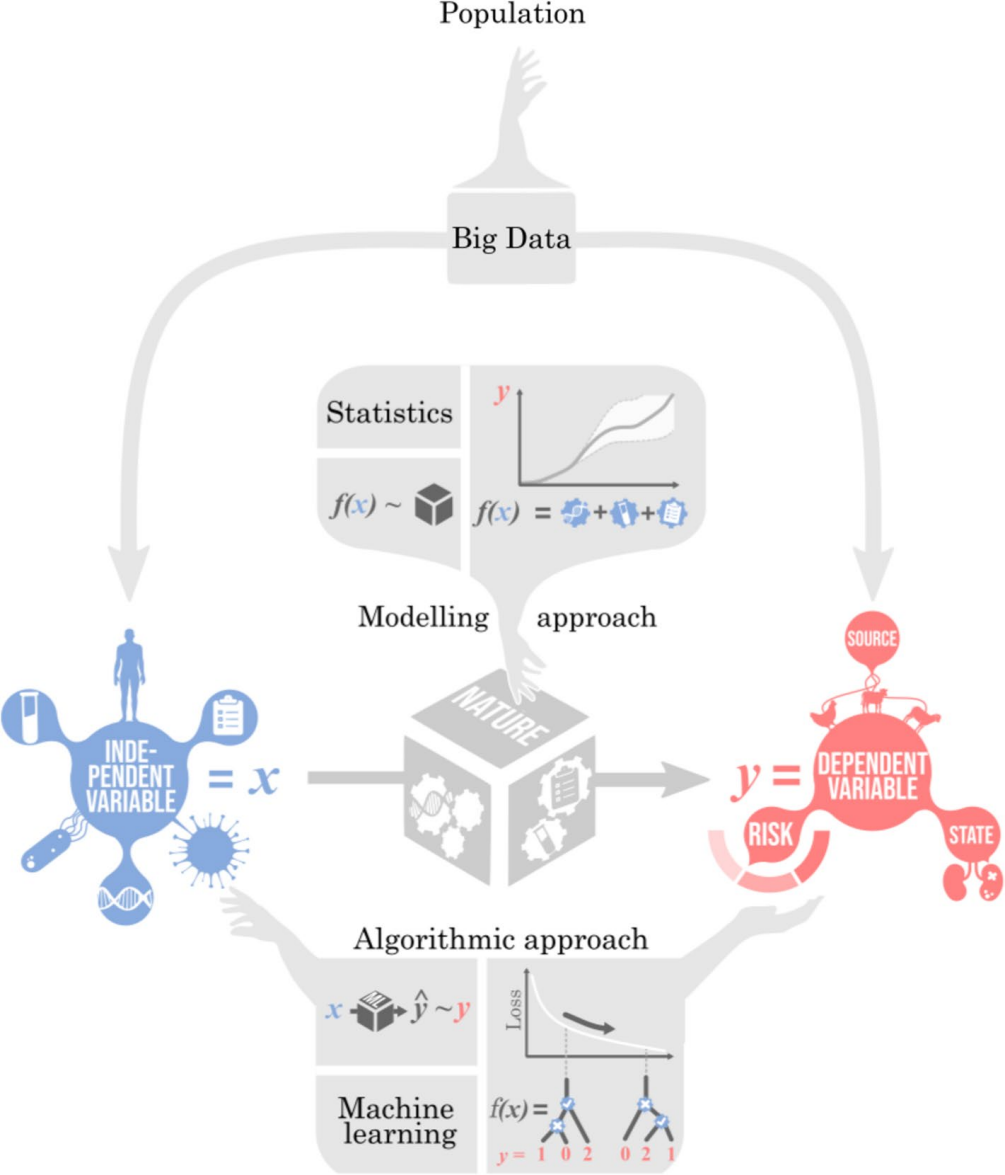
Modelling and algorithmic approaches. Big data drawn from an example population that describes the objects from which data are randomly sampled (hand). This contains features, otherwise known as independent variables, predictors or regressors, and outcomes, otherwise known as dependent variables, labels, classes, or targets, whereby changes in the features lead to changes in the outcomes. Relating the two is the data generating process, or “nature”. Statistics (or, more precisely, data modelling in Breiman’s dichotomy [12]) aims to understand the underlying processes while ML (or, more precisely, algorithmic modelling in Breiman’s dichotomy) aims to faithfully reproduce the observed patterns to achieve optimal prediction, for instance

A distinction has been drawn between competing approaches to modelling data generating processes: *data modelling* and *algorithmic modelling* [12] (Fig. 1).

Traditionally, data modelling has been the dominant paradigm, particularly in statistics, in which the data generating process is approximated by deriving a model based on assumptions about relationships between variables, both deterministic and stochastic. Data modelling emphasizes interpretability of the model and transparency of modelling assumptions. Model complexity is usually chosen by trading off realism against tractability, considering parsimony and computational burden. Special emphasis is often placed on *domain-specific knowledge* and *probabilistic models* in the data modelling approach. However, models need not be complicated, with simple additive linear assumptions underpinning workhorses like linear regression, logistic regression, and ANOVA.

In contrast, algorithmic modelling aims to provide general purpose approximations to unknown data generating processes without detailed prior knowledge [13]. Recent advances in ML have focused attention on algorithmic modelling, which also encompasses non-parametric statistical techniques. It leans on flexible algorithms capable of accurately reproducing the structure of complex data in very general settings. This flexibility often entails parameter-rich models that require large training datasets. Therefore algorithm development in ML prioritizes *computational efficiency*. Deep neural networks have proved particularly adept for algorithmic modelling [14]. In addition, the ML toolkit comprises a wide variety of techniques, many of which are available in *Python* software libraries such as Scikit-Learn, PyTorch, and TensorFlow [15–17].

### Supervised vs unsupervised learning

In *supervised learning*, a mathematical function models the relationship between variables representing the *features x* and the *outcomes y*, often with the aim of explaining or predicting *y* in terms of *x*. Very often *y* is low-dimensional; it can be binary, e.g., describing if an event does or does not occur, categorical, describing one of several possible outcomes, or continuous. In contrast, *x* is often high-dimensional, consisting of many inputs that potentially influence or predict the outcome of interest. In microbial population genomics, *y* would often be a phenotype, such as drug susceptibility, and *x* could represent the genome sequences. Genomes are typically encoded numerically for such analyses as described below. Supervised learning includes familiar approaches such as *classification* and *regression* for modelling genotype-to-phenotype relationships (e.g., [18, 19]). In *unsupervised learning*, a mathematical function models the relationship within the data *x*, often to reveal hidden structure or simulate new data. In recent approaches, such as the large language models (LLMs) that power ChatGPT, *x* represents digitally encoded text numbering several hundred billion words [20]. In microbial genomics, an important application of unsupervised learning is the detection of genetic clustering (e.g., [21–24]).

### Feature engineering for genome sequence data

Before analyzing genome data, the molecular sequences must be encoded as *features*, or vectors of numbers. Typically, features are defined in terms of genetic variation—the parts of the sequence that differ between two or more genomes. Features are often defined relative to a reference genome. For example, single-nucleotide polymorphisms (*SNPs*), which may be encoded as elements of a binary vector representing the reference allele (e.g., by the number 0) or non-reference allele (1) in each genome. If more than one non-reference allele exists, the second and third non-reference alleles are represented by additional binary vectors, so a single SNP generates multiple features, known as *dummy variables* or *one-hot encoding*. Likewise, *alleles* at a specific locus can be represented with binary vectors recording the presence (1) or absence (0) of each non-reference sequence in each genome. If there are *K* alleles at a locus, this produces *K − *1 features. For accessory genes, *presence or absence* of the entire locus can be encoded as a binary vector.

Reference-free approaches are also popular. Genome assemblies or protein sequences can be chopped into short, overlapping windows of oligonucleotides or oligopeptides known as *k*-mers, where *k* represents the sequence length. The presence or absence of each *k*-mer in each genome can be encoded as a binary vector. Very short *k-*mers (*k* < 5) are informative about nucleotide composition, whereas *k*-mers in the range 10–50 can capture locus-specific variation in SNPs, indels, and gene presence or absence. If *k* is much longer, the *k*-mers become rare or unique to individual genomes, and thereby less useful for inference or prediction. More advanced uses of *k*-mers reduce the number of features while retaining their biological meaning, for example by merging *k*-mers that always (or sometimes) share the same (or similar) pattern of presence versus absence across genomes into a smaller number of *unitigs* (or *embeddings*) which are encoded as binary (or continuous) vectors (e.g., [24, 25]).

## Biological questions and analysis goals

Framing the goal of an analysis firmly in terms of the biological question helps narrow down the appropriate ML or statistical inference tools. Biological questions map on to analysis goals including (i) data exploration, (ii) prediction, (iii) parameter estimation, and (iv) hypothesis testing. In *data exploration*, the aim is often familiarization, visualization, or hypothesis generation. These aims are open-ended, but they share a common theme of identifying or communicating important characteristics of the data or—conversely—of not missing important aspects of the data. Often, an analysis goal can be formalized quantitatively through a *loss function* that is constrained or minimized. Considering loss functions helps when comparing ML and statistical approaches.

In *prediction*, the aim is to predict, impute, classify or simulate new, unobserved, or deliberately masked data by exploiting patterns in observed data, while simultaneously minimizing prediction error: the difference between the truth and the prediction. Common loss functions for prediction include squared error for continuous outcomes or 0–1 mis-classification error for discrete outcomes, where 1 indicates misclassification and 0 indicates correct classification [26, 27].

In *parameter estimation*, the aim is to precisely quantify the parameters of a mathematical model assumed to describe the data generating process. Common loss functions for estimation include the error, absolute error, and squared error. Finally, in *hypothesis testing*, the intention is to draw qualitative conclusions, for example that a variable affects the outcome. Here, false positives are commonly encoded as a 0–1 loss function indicating whether a null hypothesis has been rejected erroneously (1) or not (0).

### Comparing performance

The performance of ML and statistical methods can be compared by averaging the loss function across observed datapoints (*empirical risk*), or across a prior distribution (*Bayes risk*), or across theoretical re-enactments of the data generating process (*frequentist risk*). Empirical risk is convenient, but requires a *ground truth*, and therefore is most applicable to prediction, where predictions might be compared directly to observed data that were deliberately masked or set-aside to measure prediction accuracy. ML offers a rich toolbox of flexible algorithms for prediction, which often helps the analyst achieve a smaller empirical risk than using traditional statistical approaches alone.

Statistical methods help when a ground truth is unavailable, for example when estimating parameters and testing hypotheses about unobserved processes. *Maximum likelihood estimation* and *likelihood ratio tests* are widely used classical approaches that minimize or constrain frequentist risks such as the mean squared error (for estimation) and family-wise error rate (for hypothesis tests). These guarantees are subject to technical assumptions like large sample size and (for hypothesis tests) nesting of models. When we are willing to make prior assumptions about the likely values of unknown parameters, Bayesian inference is useful for parameter estimation and hypothesis testing, because it minimizes or constrains Bayesian loss functions like the mean squared error (for prediction or estimation) and false discovery rate (for hypothesis tests). It does not rely on assumptions like large sample size, but Bayesian approaches can be computationally intensive.

### Fitting models

**Fig. 2:**
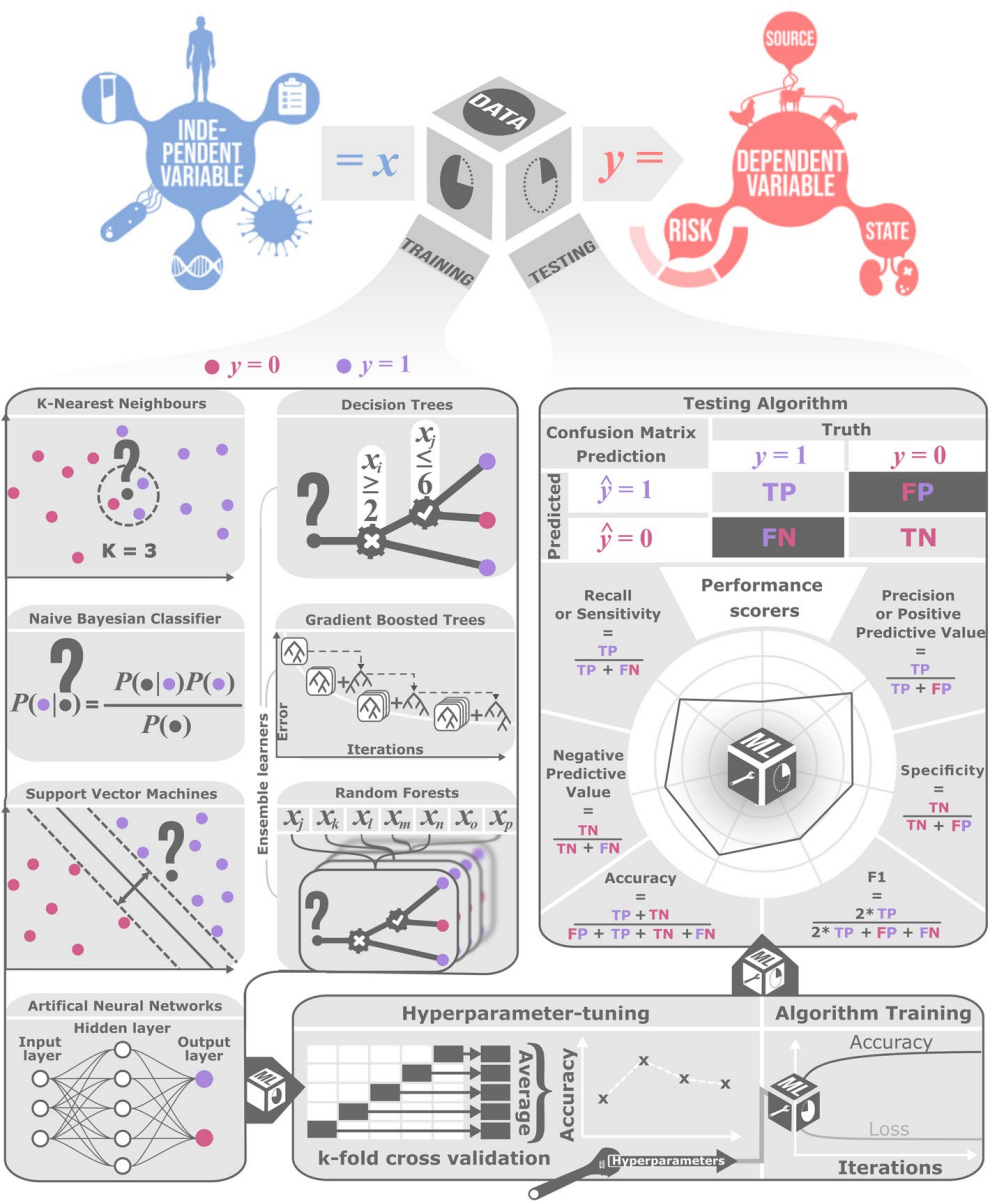
Machine learning workflow in classification tasks. The data is split into training and testing, after which a suitable general-purpose algorithm is chosen, its hyper-parameters tuned and fitted to the training data. The performance of the fitted classifier is subsequently measured using a metric of choice

Data are typically split into *training and testing* sets when a ground truth is available, allowing empirical risk to be minimized (during training) and measured (during testing). Parameters are optimized using training data, then final performance is evaluated using testing data. The idea is to obtain an independent, unbiased estimate of performance, but this may be undermined by dependence between training and testing data. Sometimes ML models entail hyper-parameters that are difficult to fit during training, so an intermediate *validation* set is employed to optimize them using grid search (Fig. 2). *Cross-validation* is a popular technique used to average over different ways of splitting the data [28]. In classical and Bayesian statistics, for estimation and hypothesis testing, often the whole data is used to fit the model, since Bayes risk or frequentist risk can be optimized theoretically. This makes more efficient use of the data.

In both ML and statistical inference, particularly in parameter-rich or data-limited settings, *over-fitting* risks noisy parameter estimates and poor generalizability to other data [29, 30]. To mitigate over-fitting, it is common to practice *regularization*, in which parameter values are constrained in some way. Examples of regularization include penalized likelihoods and Bayesian priors. *Ensemble methods*, like bootstrap aggregating in Random Forest and boosting in Gradient Boosted Trees, reduce over-fitting by optimizing performance across pseudo-replicated data. In contrast, *dropouts* in artificial neural networks avoid over-fitting by optimizing performance across randomly pruned networks to build resilience and avoid unstable over-specialization of neurons during training. Training algorithms in ML can often be tuned to reduce over-fitting by modifying a tuning parameter known as the *learning rate*, and developing strategies known as *early stopping rules*. Concerns about over-fitting must be weighed against over-correcting by poorly or under-fitting the model, a balance known as the *bias-variance trade-off*.

### Machine learning classifiers common in microbial genomics

In *classification*, the challenge is to predict or explain the outcome variable, *y*, a categorical variable (or “class”) that takes one of a fixed number of values (or “labels”), using information in the features, *x*. Typically, algorithms have parameters that are calibrated by optimizing accuracy in a training dataset. There are several common ML classifiers used in microbial genomic analyses with varying levels of complexity. Among the earliest classification algorithms is *k-Nearest Neighbors*. Here, the inferred class is the one most frequently observed in the *k* datapoints from the training data that are closest to *x*, in some sense. This requires a distance metric [31, 32]. Applications include predicting gene function and phenotypes from DNA sequences [33, 34]. Another relatively simple method is the highly scalable *Naïve Bayes* approach, where the class is assigned using Bayes’ theorem assuming independence among the features. Here, the inferred class is the one with the highest posterior probability [35–37]. Statistical distributions (e.g., Gaussian, Bernoulli) are assumed for conditional likelihoods, whose parameters must be learned. Applications include disease diagnosis [38] and sequence-based taxonomy for genomes, meta-genomes [39], and horizontally transferred genes [40].

There are several more sophisticated approaches including Support Vector Machines, Decision Trees and Artificial Neural Networks. *Support Vector Machines* offer a flexible approach to classification based on kernels, which measure the similarity of features between datapoints. Non-linear kernels facilitate classification in difficult problems like image analysis. Results can be sensitive to tuning the parameters [41–43]. Applications include detection of horizontal gene transfer [44], predicting molecular phenotypes from genome sequences [45, 46] and classifying host specificity [47].

*Decision Trees* can be compared to keys in biology field guides used to identify species. Here, decision trees represent a hierarchical sequence of rules that use features to assign a label or class. Rules are trained using heuristic “greedy” algorithms and pruned to mitigate over-fitting. Easy-to-interpret, individual decision trees are usually used ensemble to improve accuracy and reduce noise [48, 49]. Well-known *Random Forests* are an ensemble method where features and datapoints are repeatedly subsampled (“bootstrapped”) when training to build many decision trees. The most frequent classification across trees is used (“aggregated”), which improves accuracy [50–52]. Applications include predicting pathogenicity, disease status, antimicrobial resistance, genome content, and host specificity [53–62]. *Gradient Tree Boosting* is another ensemble method in which a forest of decision trees is grown stepwise, with the last tree trained to improve the previous step’s prediction, assessed via a loss function [63–65]. Applications include predicting pH preference and antimicrobial resistance from relevant gene sequences [66, 67].

Finally, inspired by neuroscience, *Artificial Neural Networks (ANNs)* have become a popular ML approach in microbial genomics. ANNs comprise directed graphs (networks) of simple functions (artificial neurons). ANNs vary in architecture but typically organize neurons into observed (input and output) layers and one or more hidden layers [68]. Communication occurs between layers of the ANN [69]. *Deep learning* employs ANNs with multiple hidden layers, which produces complex and flexible models with large information processing capacity [70, 71]. Advances in big data availability, GPUs (graphics processing units) and theoretical innovations have allowed parameter-rich ANNs to be fitted efficiently. Applications include identifying species, strains, and gene function from DNA sequences [72–75]. ANNs perform well partly because they act as universal function approximators through approximating arbitrary continuous relationships, given enough hidden neurons [76], and partly because the fitting techniques are thought to impose regularization (e.g., [77]). *Attention mechanisms* enable some ANNs, notably *transformers*, to dynamically weight the influence of input elements based on context, rather than relying on fixed connection patterns [78]. This allows the network to selectively focus on the most relevant parts of the input, regardless of their position. Attention is useful for dependencies in molecular sequences or three-dimensional protein structures, where traditional architectures struggle to propagate long-range information. Attention mechanisms allow every input element to directly consider all others in parallel, avoiding the dilution of important but distant signals. Attention has driven breakthroughs in generative AI [79], antibiotic prediction [7, 8], and protein structure prediction [5].

## Strengths and weaknesses of machine learning and statistics

A clear statement of the biological questions informs the analysis goals by determining what type of loss to minimize. Minimizing estimation error versus prediction error versus false positives guides the choice of method. Data analysis aimed at understanding underlying processes causally is better served by statistical inference, because it will minimize the (Bayesian or frequentist) risk associated with estimation and hypothesis testing. Data analysis aimed at optimizing model-agnostic problem-solving performance is better served by ML, because it can minimize the (empirical) risk of prediction whenever a ground truth is available [29, 80]. The dominant statistical paradigm emphasizes principles like *parsimony* and *explainability*, whereas sophisticated ML algorithms can produce demonstrably superior performance over simpler models common in statistics. This is exemplified by classic supervised learning examples like the XOR problem, where the output is not a linear function of the input data.

Out-of-the-box, many ML approaches deal with *collinearity*, *non-linearity*, and *interactions* better than traditional statistical approaches like regression. An experienced statistician might employ regularization to counter unreliable parameter estimates and high uncertainty caused by strongly correlated or collinear features, but regularization is built in to many ML algorithms as standard. Non-linear relationships between features and outcomes, and interactions between features, can also be modelled statistically, but this requires some sophistication and manual intervention on the part of the data analyst, whereas many ML algorithms are designed to model these phenomena automatically. ML algorithms can often prioritize among thousands of features, allowing the user to take an agnostic approach to feature selection. However, the cost of sophisticated ML is models whose workings and parameters are less transparent to interpretation [81], often termed *black boxes* [19].

The strong performance of machine automation and the advantages of model agnosticism have de-emphasized the perceived importance of human accountability for *data quality* issues; this is known as *automation bias*. *Biased sampling* and *batch effects* create problems for both ML and statistical inference, by generating conclusions that may be misleading or poorly generalizable (see “Data quality and interrogating results”). Moreover, concerns relating to *interpretability*, *equality*, and *accountability* are important in many settings, particularly healthcare [82]. Therefore, trade-offs that exist between the performance of a model in a specific, loss function sense, versus its wider utility for society, may shift the balance between preferring ML versus statistical inference. The ML vs statistics and data modelling vs algorithmic modelling dichotomies recall a more fundamental distinction between *deductive* (logic-based) vs *inductive* (observation-based) scientific inference. The fundamentally *empirical* approach to modelling of ML is data-driven and data-hungry, explaining its reliance on big data and susceptibility to biased datasets, but also its superior flexibility to fit the data more closely.

### Data quality and interrogating results

The adage “garbage in, garbage out” is a truism in ML and statistics: proper data preparation and quality checking (*QC*) are indispensable to any analysis. Researchers must adopt strategies to diagnose *data quality* issues before and after analysis.

As a first step, it is essential to understand the *provenance* of the data, its limitations, and whether it is adequate for the analysis goals. Next, data must be quality-checked using approaches including summary statistics and visuals to diagnose issues such as data entry errors, outliers, missing values, and special values. Data must be properly encoded, especially missing or special values, to ensure ML or statistical algorithms handle them appropriately. An *imputation* step, to predict missing values, may be required. Alongside QC, data exploration is valuable for hypothesis generation and selecting an appropriate model that makes reasonable assumptions.

Before merging datasets that may have been collected in different places, at different times, by different processes, or for different purposes, it is important to consider how the analysis might be influenced by *heterogeneity*—systematic differences between the datasets. For example, there might be unmeasured *confounders* that differ between them. Systematic differences in outcomes across datasets make analysis particularly vulnerable to so-called *batch effects*. Sometimes heterogeneities are “controlled” by including the batch label as a feature. A more robust but less efficient approach is *meta-analysis*, in which datasets are analyzed separately and results are compared post-analysis, merging if appropriate. Often this fits neatly into training, testing, and validation, especially since *out-of-sample prediction* is a more robust indicator of generalizability than splitting a single dataset.

After analysis, it is essential to interrogate the data again to understand where informative signals come from and diagnose unresolved QC issues or implementation *bugs*. A healthy *skepticism*, especially for surprising results, is important and consideration of questions such as: (i) How do results compare to the *literature*? (ii) Are results robust to the analysis assumptions? Benchmarking against simpler methods can help here. Unless the signal in the data can be explained—for example using visualization or explainable AI—it may be difficult to convince peers. Experimental *validation* and *replication* in independent datasets are often required to build credibility and, to repeat another truism, “extraordinary claims require extraordinary evidence.”

## Applications of ML and statistics in microbial genomics

In this section, we consider applications of ML and statistics in microbial genomics and discuss the relative advantages of the competing approaches in the context of three examples: source attribution in zoonotic bacteria, genome-wide association studies of antimicrobial resistance, and predicting antimicrobial resistance from genome sequences.

### Example 1: Source attribution in campylobacter

#prediction #classification #supervised_learning #machine_learning.

Features (***x***): genome sequences. Outcomes (***y***): host species of origin.

Identifying the population-of-origin of bacterial infections has practical applications for a range of pathogens, particularly multi-host organisms that cause zoonotic infections in humans like *Salmonella*, *Escherichia coli*, and *Campylobacter*. Person-to-person transmission of *Campylobacter*, a common cause of gastro-enteritis in humans, is rare, with most cases caused by consumption of contaminated food. *Campylobacter* commonly colonizes the guts of birds and mammals, including animals farmed for meat and poultry, and is found in environmental water. Therefore, each human case is thought to originate from one of the source reservoirs, and it is useful to predict, or “attribute,” the source. Source attribution helps prevent future human cases by informing efforts to disrupt the transmission chain.

DNA sequencing has been exploited for source attribution in *Campylobacter* using a variety of tools. Data typically comprise DNA sequences of *Campylobacter* isolated from cases of human infection, and, for comparison, from animal and environmental reservoirs. Early approaches pursued statistical epidemiological models, using strain-level designations to rule out transmission (e.g., [83, 84]). Later, statistical models grounded in population genetics, like *Structure* and *iSource*, were applied, which exploited more of the information in the DNA [85, 86]. However, source attribution can be formulated as a straightforward ML problem, where the analysis goal is to minimize mis-classification error. DNA sequences of *Campylobacter* sampled directly from source populations can be used to train a classifier with known labels (e.g., cattle, sheep, pigs, chicken, environmental water). Classifier accuracy can be tested using cross-validation. The population-of-origin of each human case can then be predicted from the DNA sequence. ML classifiers proved to be faster and around 11% more accurate than the established statistical approaches applied to multi-locus sequence typing (71% vs 64%), and readily generalized to the analysis of whole genome sequencing (WGS) data, permitting 33% gains in accuracy (85% vs 64%) [58, 59]. Random Forest [51] and XGBoost [87] produced the greatest improvements. Key to the success of ML in this context has been the availability of big data comprising thousands of whole genomes with a high degree of replication: 5799 genomes sampled from the source populations of interest, together with 15,988 genomes from human infection.

### Example 2: genome-wide association studies of antimicrobial resistance

#hypothesis_testing #parameter_estimation #regression #statistics.

Features (***x***): genome sequences. Outcomes (***y***): antimicrobial resistance or sensitivity.

A major aim of biology in the twenty-first century is to unravel the genetic architecture of phenotypic diversity within species [88]. In microbiology, there is particular interest in traits that affect the outcome of human colonization and infection, like virulence (the frequency or severity of disease) and antimicrobial resistance (AMR). Early approaches to such questions studied candidate genes, for example using PCR to test for differences in the frequency of genetic markers between cases and controls (e.g., [89]). With the advent of technologies like genotyping arrays and, later, whole-genome sequencing, the accepted approach to such questions has been to scan the genome for evidence of associations between allelic differences and phenotypic differences. So-called genome-wide association studies (GWAS) address concerns that candidate gene approaches are vulnerable to selection and reporting bias, and struggle to control artefactual associations caused by population stratification of phenotypes, for example when phenotypes differ between strains [90–92].

GWAS is motivated by a desire to learn about the causal process underlying the data, and pains are taken to avoid artefactual signals of association, while recognizing that observational studies cannot prove causality (see, e.g., [93, 94]). This is a statistical inference problem in which the parameters of a relatively simple and readily interrogated general linear model are interpreted to identify genetic variants responsible for observable phenotypic diversity. Special emphasis is placed on limiting the expected losses caused by false positive associations. In bacteria, GWAS have been applied to a range of traits and species (e.g., [54, 95–99]). While ML approaches have been applied to this problem, and while informative for data exploration and hypothesis generation, particularly in expert hands [100], ML approaches only return “high-leverage” genes or genetic variants that help predict the outcome. Out-of-the-box they neither test nor quantify the evidence for the hypothesis that these variants directly influence the outcome. Nor do they offer theoretical or empirical tools for easily controlling family-wise error or false discovery rates across loci. Statistical approaches address these foundational issues, and mapping of genes underlying AMR has proved particularly fruitful (e.g., [101, 102]), presumably because mechanisms of genetic resistance are often direct, almost deterministic. GWAS depends on big data to find signals of association, but interpretation of those signals relies on explicit modelling assumptions, and not on training a general-purpose algorithm using datasets of many known genotype-to-phenotype associations, which as yet do not exist.

### Example 3: predicting antimicrobial resistance from genome sequences

#prediction #classification #supervised_learning #interpretable_machine_learning.

Features (***x***): genome sequences. Outcomes (***y***): antimicrobial resistance or sensitivity.

Related to the problem of inferring which genes confer antimicrobial resistance is the problem of predicting antimicrobial resistance from an individual bacterial genome. Modernizing microbiological diagnostics in clinical practice has been a major focus of research over the last 15 years, with aspirations to replace a battery of phenotypic tests with a streamlined WGS and phenotype prediction pipeline [103]. WGS has become routine in some healthcare settings, particularly for organisms that are challenging to test in the laboratory, like the slow-growing and high biosafety level pathogen *Mycobacterium tuberculosis* [104, 105].

The statistical models used for GWAS could be turned to prediction, but the superior flexibility of ML algorithms to fit data more closely make them a natural choice for predicting AMR (e.g., [100, 106–110]). In this setting, the analysis goal is to minimize prediction error, which can be quantified empirically because a ground truth is available. Large datasets have been generated comprising WGS and traditional AMR phenotyping assays and based on these, automated predictions with high accuracy have been achieved—in some cases exceeding the standards required of traditional laboratory diagnostics [111, 112]—confirming the excellent performance of ML algorithms for general-purpose prediction.

ML performance in AMR prediction has established it as an important tool for predicting all manner of bacterial phenotypes from WGS data. However, there is a question of accountability: in a medical setting, decision-taking responsibility lies with the clinical microbiologist. Therefore the ML algorithm needs to present the evidence for its prediction transparently for interpretation by the domain-specific expert. Scenarios like this create a need for *explainable AI* that goes significantly beyond outputting coefficients for predictive features, which may be mere confounders, rather than biologically causal genetic variants, particularly in the presence of population stratification [113]. Approaches to explainable AI include *attribution algorithms*, which may impose post hoc linearization of the predictions (e.g., [114, 115]). This leads back to simpler, more transparent data models resembling additive or linear models. Alternatively, *ablation algorithms* systematically drop features-of-interest from the model to assess their impact on performance [116]. Consequently, even when pursuing prediction via complex ML, efforts to interpret prediction may resemble more traditional statistical analysis in which high importance is attached to understanding in a causal way the conclusions and interpretation of the data.

## Statistics versus machine learning: right tool for the right job

The boundary between ML and statistics is blurred, with cross-over methods like the elastic net, bootstrap, non-parametric statistics, and Bayesian-inspired approaches. The labels “machine learning” and “statistics” are typically less useful than a clear definition of the analysis goals—prediction, exploratory data analysis, parameter estimation, hypothesis testing—which in turn are framed by the biological questions. Where a project has multiple goals, such as prediction and hypothesis testing, it is reasonable to apply different analysis approaches to the same data. However, as example 3 illustrates, even when a task clearly fits the goal of prediction, the choice of method is influenced by context-specific considerations, notably explainability and accountability. Frequently in scientific applications, there is an emphasis on understanding and interpreting the data generating process, and this may tip the balance away from ML and towards statistical inference. Interrogating results in real data analysis, detecting data quality issues like batch effects, explaining which signals drive the results, controlling for confounding factors, and understanding the limits to generalizability, are essential to the integrity of scientific outputs. Developing strategies to check scientific results is a key step towards scientific independence that allows a researcher to take responsibility for final conclusions. The risk of automation bias, in which responsibility for final conclusions is delegated to opaque algorithms, and abdication of critical thinking, are rightly of concern.

### Conclusions and future directions

We are currently in a period of exploration, as ML and AI are increasingly applied to diverse questions like “what is the genetic architecture of virulence,” “why do dangerous pathogens emerge,” and “how do we fight the spread of antimicrobial resistance”? In allied fields, we have seen transformative innovations ranging from the prediction of 3D molecular structure [5] to antimicrobial peptide discovery [7, 8] and, looking ahead, the design of novel proteins and molecular systems based on free text (e.g., [117–120]). In microbial population genomics, we anticipate that ML will continue to play a leading role, both by improving on previous approaches, and by opening new avenues of research and understanding. If, in the years to come, there were to be a final analysis of the role of ML and AI in microbial genomics, no doubt it would re-emphasize the enduring importance of deductive statistical thinking, currently less fashionable as the new opportunities presented by ML take precedence. Statistics provides a foundation for scientific thought, clarifying concepts like study design, randomization, replication, control, batch effects, mediation and confounding, causation, and correlation. Scientific progress is a continual process, so there will be no final analysis. Instead, we expect a gradual assimilation of recent developments in AI/ML together with well-established statistical approaches into a new and emerging field of Data Science.

### Glossary 1: Terms in statistical inference and machine learning, generated by ChatGPT-4o and manually curated

#### Attribution algorithms

Methods that assign importance scores to input features by estimating their contribution to a model’s prediction, often using gradients, perturbations, or local surrogate models (e.g., SHAP, LIME).

#### Ablation algorithms

Techniques that assess feature importance by systematically removing or masking input features and measuring the resulting impact on model performance or predictions.

#### Automation bias

The tendency for humans to over-rely on automated systems, such as ML models, even when they may be incorrect.

#### Batch effects

Non-biological variations introduced into data during different processing times, instruments, or sample batches, which can confound results.

#### Bias-variance trade-off

A fundamental concept that describes the balance between underfitting and over-fitting. High bias models are too simple and may miss patterns (underfitting), while high variance models are too complex and may capture noise as if it were signal (over-fitting). Optimal performance is achieved by balancing these two sources of error.

#### Biased sampling

Occurs when the sample used for training or testing a model is not representative of the overall population-of-interest, leading to biased, misleading, or non-generalizable results.

#### Black box

A term used to describe models (such as deep neural networks) that are complex and difficult to interpret, where the internal workings are not easily understood.

#### Computational efficiency

Refers to the amount of computational resources (time and memory) required to train and use a model. More efficient models can handle larger datasets or run faster.

#### Collinearity

Collinearity occurs when two or more features are highly correlated, meaning they share a linear relationship. This makes it difficult to estimate the unique contribution of each predictor, leading to unreliable estimation with high uncertainty.

#### Cross-validation

A technique for assessing how well a model generalizes to unseen data by partitioning the dataset into multiple subsets and training/testing the model on different combinations of these subsets.

#### Data generating process

The scientific and sampling mechanisms by which the observed data in a study were produced.

#### Data quality

Refers to the accuracy, completeness, and reliability of data, which directly affects the performance of statistical inference and ML.

#### Data vs algorithmic modelling

In Breiman’s dichotomy, *data modelling* focuses on building models that capture the essence of the underlying data generating process, whereas *algorithmic modelling* focuses on flexible prediction algorithms that exploit the structure in the observed data, without making assumptions about the underlying data generating process.

#### Deep learning

A subset of ML that involves neural networks with many layers (deep architectures) used to model complex patterns in data, particularly useful for image, speech, and sequence tasks.

#### Deductive vs inductive reasoning

*Deductive reasoning* draws specific conclusions from general principles or theories, whereas *inductive reasoning* infers general patterns or rules from specific observations or data.

#### Domain-specific knowledge

Expert knowledge about the particular field or domain of application (e.g., genomics) that helps guide model development and interpretation of results.

#### Dropouts

A regularization technique commonly used in neural networks where random units (artificial neurons) are “dropped” or ignored during training to prevent over-fitting.

#### Early stopping rules

A technique used to stop training a model once its performance on a validation set starts to degrade, preventing over-fitting.

#### Empirical

Based on observation or experimentation rather than theory. Empirical data is gathered from real-world experiments or observations.

#### Ensemble methods

ML methods that combine the predictions of multiple models to improve accuracy and robustness. Common examples include Random Forest and Gradient Boosting.

#### Explainability

The ability to interpret and understand how a ML model makes decisions, particularly in complex or high-dimensional models.

#### False discovery rate (FDR)

The expected proportion of false positives among all rejected null hypotheses in multiple hypothesis testing.

#### Family-wise error rate (FWER)

The probability of making one or more false positive errors when performing multiple hypothesis tests.

#### Features

Individual measurable properties or characteristics of the data used to train a model; they are analogous to independent variables in traditional statistical terminology, representing the inputs used to predict or explain an outcome.

#### Hypothesis test

A statistical method used to determine whether there is enough evidence to reject a null hypothesis, usually based on the comparison of a test statistic to a critical value.

#### Interactions

Interactions occur when the effect of one feature on the outcome depends on, or is modified by, the value of another feature.

#### Interpretable machine learning

A branch of ML focused on developing models that provide human-understandable explanations for their predictions.

#### Interpretability, equality, and accountability

Important ethical considerations in ML that refer to the clarity of model outputs (*interpretability*), fairness across different groups (*equality*), and responsibility for decisions made by models (*accountability*).

#### Learning rate

A hyper-parameter that controls how much a ML model’s weights are updated with respect to the gradient of the loss function during training.

#### Loss function

Quantifies the (lack of) quality of a model’s performance relative to the biological aims. It guides the optimization process during training. Examples include the mean squared error, calculated between a prediction or estimate and the truth, and 0–1 loss, where a value of 1 indicates a misclassification error or a false positive. Usually it is *risk*, rather than loss, that is minimized.

#### Maximum likelihood estimate (MLE)

A method of estimating the parameters of a statistical model by maximizing the likelihood function, which measures how likely it is to observe the given data under different parameter values.

#### *Maximum *a posteriori* estimate (MAP)*

An estimation method that incorporates prior knowledge or beliefs about the parameters in addition to the likelihood of the data, often used in Bayesian statistics.

#### Non-linearity

Non-linearity refers to relationships between variables that cannot be adequately captured by a straight line. In a non-linear relationship, changes in one variable do not simply lead to proportional changes in another.

#### Outcomes

The target variables that a model aims to predict or explain; they are analogous to dependent variables in traditional statistical terminology, representing the outputs that depend on the input features.

#### Over-fitting

Occurs when a model learns not only the underlying pattern in the training data but also the noise, leading to poor performance on new, unseen data.

#### Parsimony

A principle that prefers simpler models over more complex ones when both explain the data equally well, often used interchangeably with Occam’s Razor.

#### Probabilistic models

Models that incorporate uncertainty by assigning probabilities to different outcomes, useful for reasoning about uncertainty in data.

#### Python

A high-level programming language widely used in data science and ML due to its simplicity, extensive libraries and strong community support.

#### Regression vs classification

*Regression* models continuous outcomes, while *classification* models categorical outcomes, in both cases fitting observed or predicting new outcomes based on the features of other input data.

#### Regularization

A technique used to prevent over-fitting by incorporating a penalty on the parameter values into the loss function (e.g., L1, L2 regularization).

#### Risk

The expected value of a *loss function*, defined as an arithmetic mean over (i) observed datapoints (*empirical risk*), (ii) a prior distribution (*Bayes risk*), or (iii) hypothetical repetitions of the data generating process (*frequentist risk*). Usually it is risk, not loss, that can be minimized by model fitting.

#### Supervised vs unsupervised learning

In *supervised learning*, a statistical or ML algorithm is trained on labelled outcome data, whereas in *unsupervised learning*, the algorithm learns from unlabelled data, discovering patterns without explicit outcomes.

#### Training, testing, and validation

A *training set* comprises data used to fit or train a model. A *validation set* is a separate subset of data used to tune model parameters and assess performance during training, where necessary. The *test set* is another, separate set of data used to evaluate the model’s performance after training is complete.

## Data Availability

No datasets were generated or analysed during the current study.
